# Composition, Technological, and Microstructural Aspects of Concrete Modified with Finely Ground Mussel Shell Powder

**DOI:** 10.3390/ma16010082

**Published:** 2022-12-22

**Authors:** Sergey A. Stel’makh, Evgenii M. Shcherban’, Alexey N. Beskopylny, Levon R. Mailyan, Besarion Meskhi, Salikh Sh. Tashpulatov, Andrei Chernil’nik, Natalya Shcherban’, Anastasia Tyutina

**Affiliations:** 1 Department of Unique Buildings and Constructions Engineering, Don State Technical University, Gagarin Sq. 1, 344003 Rostov-on-Don, Russia; 2 Department of Engineering Geology, Bases, and Foundations, Don State Technical University, 344003 Rostov-on-Don, Russia; 3 Department of Transport Systems, Faculty of Roads and Transport Systems, Don State Technical University, 344003 Rostov-on-Don, Russia; 4 Department of Roads, Don State Technical University, 344003 Rostov-on-Don, Russia; 5 Department of Life Safety and Environmental Protection, Faculty of Life Safety and Environmental Engineering, Don State Technical University, 344003 Rostov-on-Don, Russia; 6 Department of Technological Machines and Mechanics, Tashkent Institute of Textile and Light Industry, Shakhjakhon Str., 5, Tashkent 100100, Uzbekistan

**Keywords:** mussel shell powder, modified concrete, concrete microstructure, strength characteristics, deformation characteristics

## Abstract

Reducing the negative environmental impact of a widely spread building material such as concrete is possible by decreasing the amount of cement in this composite material, especially when specific waste is included as a substitution for the binder. Another important environmental issue is accumulated aquaculture waste. This work justifies the possibility of achieving modified concrete with improved properties based on sea mussel shell powder (MSP). An improved environmentally friendly concrete was obtained and modified with MSP as a result of experimental studies. The dosage of MSP in the amount of 6% instead of part of the cement turned out to be optimal and most effective. Because of the modification, it was possible to increase the strength properties: the increments were up to 12% for the compressive strength (CS), up to 13% for the axial CS, up to 14% for the tensile strength (TS) in bending, and up to 12% for the axial TS. The ultimate strains under axial compression and tension decreased to 9% and 12%, respectively, and the elastic modulus increased to 15%. SEM analysis showed a more integral microstructure without voids and cracks in this composite with a modifier content of 6% compared with the sample of the ordinary composition. Economic efficiency is expressed in reducing the total cost of new concrete compared to traditional ones by about 17% and the cost of building construction by up to 15% due to a decrease in the percentage of defects.

## 1. Introduction

Currently, there are several significant problems in the fields of ecology and engineering. A number of these problems are directly interconnected and justified by the fact that engineers, in turn, experience a lack and shortage of environmentally friendly production methods while, at the same time, ecology and the environment suffer quite seriously [1,2,3,4,5]. In addition, there are other branches of human life, such as agriculture and aquaculture, that is, the cultivation of various river and seafood products to obtain food. However, in several specific industries, during aquaculture activities, a large amount of waste of organic origin accumulates [6,7,8,9,10,11]. Undoubtedly, one of the most acute problems is the accumulation of a large number of shells of living organisms, which are grown for subsequent consumption. Such waste includes mussel shells [12].

Thus, there is a considerable environmental problem that needs to be addressed in the most efficient way. At the same time, taking into account deficiencies in the field of engineering knowledge on building material manufacture, there is a need to develop new production technologies, create new recipes, as well as conduct fundamental research on the issues of structure formation and establish dependencies of “composition-structure-property”, that are related to the study of various components for concrete and the properties of concretes themselves in general [13,14,15].

Table 1 presents a review of the literature exploring various types of predominantly aquacultural waste being used in the construction industry and in the manufacture of various types of composites.

Following the results of this literature review [15,16,17,18,19,20,21,22,23,24,25,26,27,28,29,30,31,32,33,34], we can confidently say that shells can be used as a component in the manufacture of concrete, and in various variations as a replacement for a part of fine aggregate, as a replacement for a part of coarse aggregate, and as a replacement for a part of the binder. Almost all seashells are 95–97% calcium carbonate (CaCO_3_), “which is comparable to the CaCO_3_ content in limestone powders used in the manufacture of Portland cement” [2]. Additionally, when seashells are roasted at a temperature of 600 °C, calcium carbonate is converted into CaO. “Calcium oxide provides an increase in the set of strength and density of concrete” [2].

In general, concretes with partial substitution of cement with seashell powder wastes, which demonstrate adequate mechanical characteristics comparable to those of concrete based on conventional Portland cement, can contribute to the solution to the general problem of reducing CO_2_ emissions [35,36,37,38].

According to the results of the literature review, it was revealed that, currently, there are quite a few investigations that study the full range of the strength and deformation characteristics of concrete modified with various amounts of mussel shell powder. The dependences between the initial factor—the dosage of the modifier based on the mussel shell powder and the output parameter—and the strength and strain characteristics (SSC) of concrete modified with MSP that were firstly obtained comprised a scientific novelty. The features of the formation of the structure of new environmentally and cost-effective improved concrete for different contents of MSP are revealed.

Thus, the purpose of this study is to substantiate the possibility of obtaining modified concrete with improved characteristics based on sea mussel shell powder (MSP).

The specific objectives of this research are:

– To search for ways to rationally process mussel shells as a valuable raw material source to obtain a new generation modifier that can significantly improve the composition and structure of concrete.
– To study the SSC of modified concrete, reflecting its performance and applicability in various structures.
– To assess concrete in terms of efficiency, including a reflection on its environmental impact.

These factors lead to a complex environmental and economic effect, which comprises the rational disposal of aquaculture waste and the creation of concrete with high functional characteristics and an improved structure as its basis.

## 2. Materials and Methods

### 2.1. Materials

The primary physical and mechanical characteristics of the binder and inert materials used for the concrete mixtures’ (CM) manufacture are presented in Table 2.

In a scientific study, finely ground MSP was used as a modifying agent. Mussel shells used as a concrete modifier are collected on the shores of the seas located in the Far East, as well as in the Black Sea.

The chemical composition (CC) of mussel shells, obtained by energy dispersive spectroscopy on the crushed mussel powder, is presented in Table 3.

**Figure 1:**
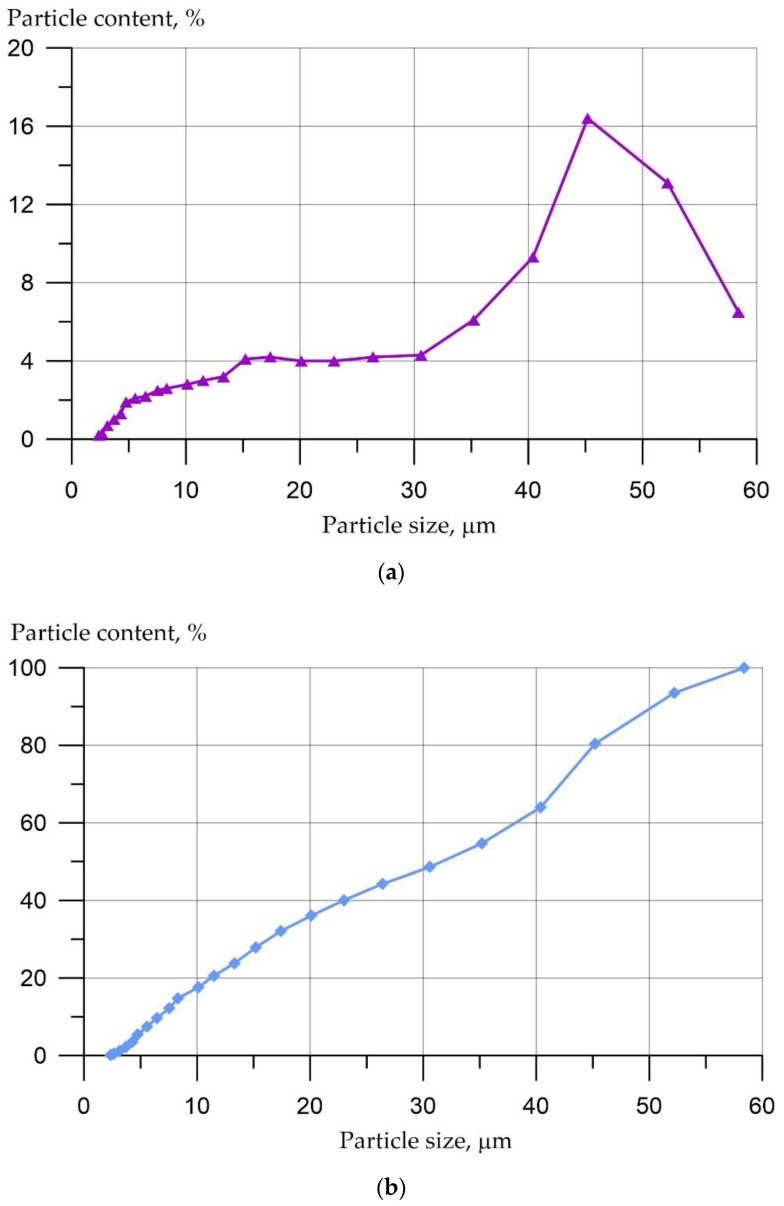
PSD curves of mussel shell powder (MSP): (**a**) PSD; (**b**) Cumulative curve.

Figure 1 shows the particle size distribution (PSD) curves of the powder obtained from mussel shells.

It can be seen from Figure 1a that the particle size of the mussel powder ranges from 2 to 59 µm. The largest proportion (about 52%) of the particles are is 32 to 59 microns in size, approximately 20% are particles from 15 to 32 microns in size, and the remaining 28% are particles from 2 to 15 microns in size.

**Figure 2:**
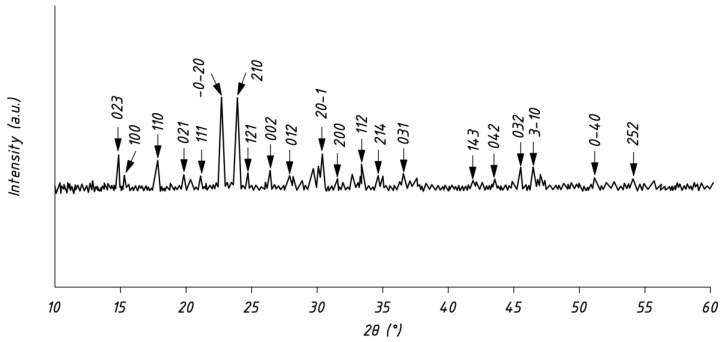
X-ray diffractometry analysis of mussel shell powder. (Intensity is diffraction intensity in a.u.—arbitrary units, 2θ is diffraction angle).

The accepted X-ray diffractometry analysis of the powder obtained from the mussel shells is shown in Figure 2.

The X-ray diffraction patterns show two sharp characteristic peaks at 2θ = 22.95 and 24.18°, corresponding to reflections (0–20) and (210) on the anorthic crystal structure, which is in good agreement with [39]. In [39], the authors performed an XRD analysis of the powders obtained from the shells of bivalve mollusks of various species (mollusks, mussels, and oysters), and the resulting X-ray patterns had two identical peaks corresponding to reflections (0–21) and (210), which is consistent with the results of this study. The authors of [39] used PDF No. 70-0090. The specified X-ray pattern, which has two characteristic peaks, was obtained in [39] by modifying and developing the surface of a fine powder of mussel shells, which is similar to our approach of preparing and developing the surface and, accordingly, activating this powder. Thus, our methodological approach is in good agreement with the methodology [39].

### 2.2. Methods

**Figure 3:**
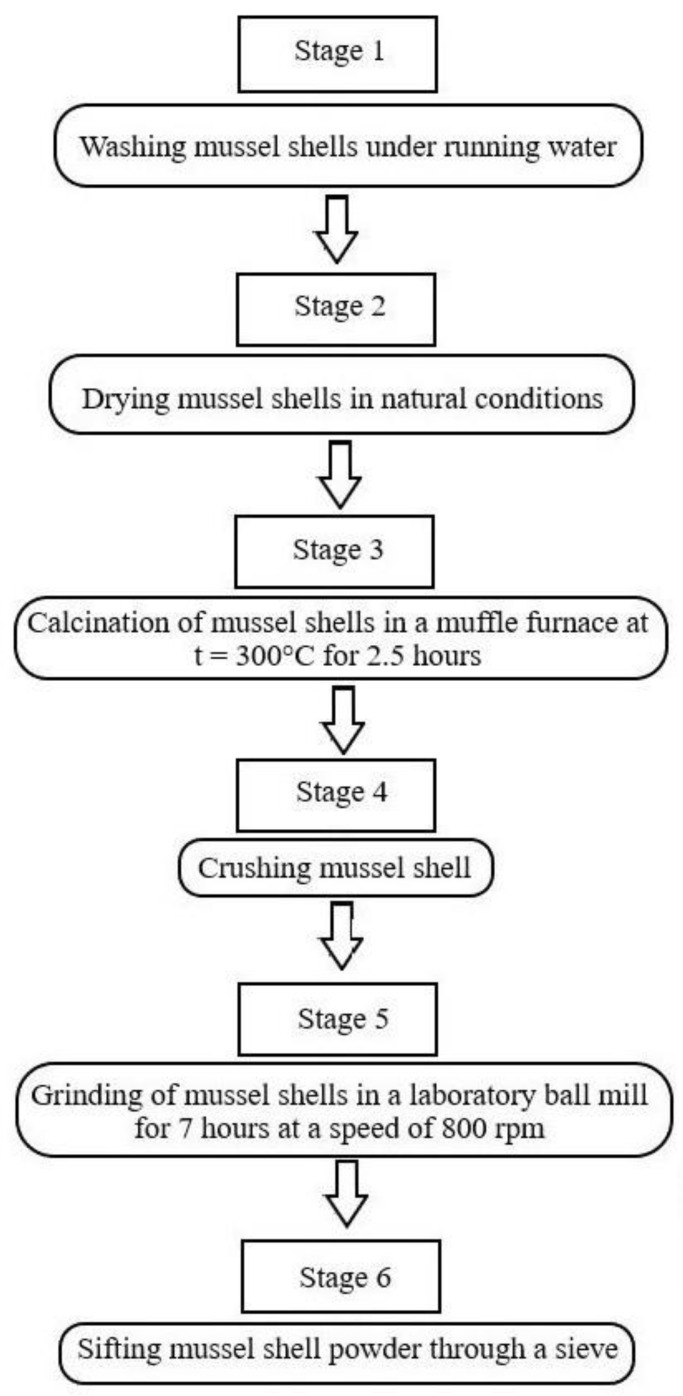
Stages of preparation of powder from mussel shells.

The main steps in the manufacture of finely ground powder from mussel shells are presented in a flowchart (Figure 3) as follows.

The thermal treatment of mussel shells at a temperature of 300 °C was carried out in order to completely remove the organic residues that remained after washing.

The design of the concrete mixture composition was carried out according to the GOST 27,006 “Concretes. Rules for mix proposing” [40] based on the actual values of the physical and mechanical characteristics of the raw materials.

In laboratory conditions, samples were made of concrete with approximately 40–45 MPa of strength, with the composition presented in Table 4.

The production of CMs, samples-cubes, and prisms with different dosages of powder from mussel shells was introduced instead of part of the cement and proceeded as follows:

– Dosing the CM components;
– Mixing Portland cement and quartz sand in a concrete mixer for 2 min;
– Adding the required dosage of MSP and mixing until a homogeneous consistency was obtained;
– Introduction of mixing water and coarse aggregate;
– Mixing of the concrete mix to a homogeneous condition;
– Unloading the resulting mixture from the concrete mixer and molding the samples;
– Smoothing the surface of the samples compacted for 1 min by vibrating and marking them [41,42,43,44,45].

The hardening of the samples took place in the normal hardening chamber for 1 day; then, the specimens were stripped and kept in a chamber under normal curing conditions for 27 days. After 28 days of curing, the specimens were tested on a hydraulic press and a tensile testing machine.

**Figure 4:**
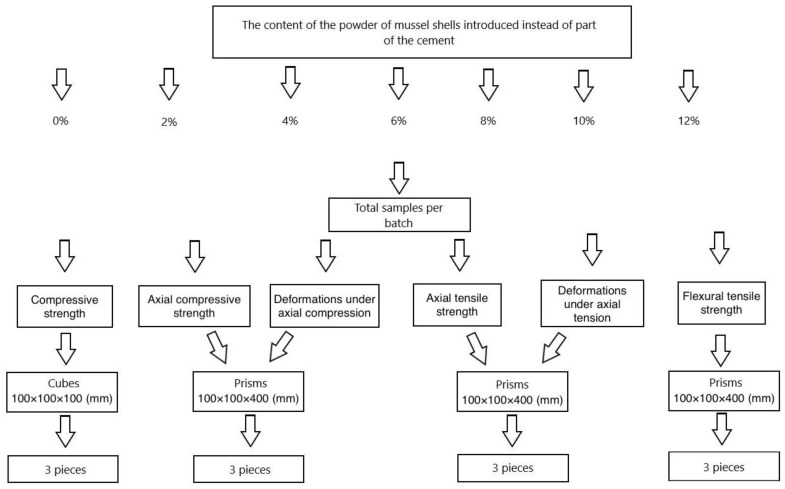
Experimental program.

Figure 4 shows the experimental program.

Determination of compressive strength (CS), tensile strength (TS) in bending, and axial TS proceeded following the GOST 10,180 “Concretes. Methods for strength determination using reference specimens” [46]. The determination of axial CS-by GOST 24,452 “Concretes. Methods of prismatic, compressive strength, modulus of elasticity, and Poisson’s ratio determination” [47].

The main technological, testing laboratory equipment, and measuring instruments for the manufacture of finely ground powder from mussel shells, concrete mixes and laboratory samples with different percentages of cement replacement by powder from mussel shells were used the same as in [41,42,43,44,45].

The microstructure of the hardened cement paste with the addition of mussel shell powder was studied using an electron microscope, which was also equipped with a special device for chemical analysis [41]. The parameters under which the SEM analysis was performed are given in Section 3, with photographs of the microstructure of the samples.

The X-ray diffraction of samples of the obtained powder was carried out on a Difrey X-ray diffractometer (JSC Scientific Instruments, St. Petersburg, Russia) with Cu K radiation operating at 40 kV and 40 mA. The diffraction angle was continuously scanned from 10° to 60° in 2θ at a scanning rate of 2°/min.

## 3. Results and Discussion

Table 5 presents the actual values of the concrete density of all experimental compositions.

Based on Table 5, there is a slight increase in the density of concrete with an increase in the dosage of MSP from 0 to 6%, then with a further increase in the amount of MSP from 6 to 12% a decrease in the density of concrete was noted.

**Figure 5:**
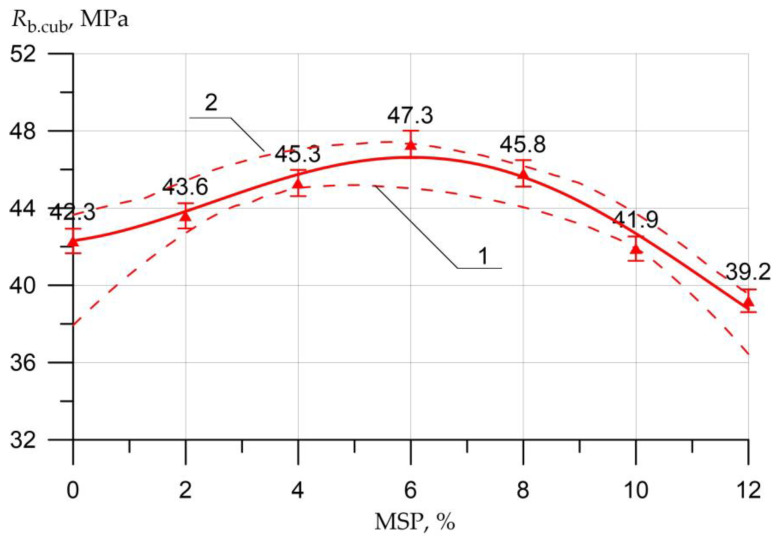
Dependence of the CS of concrete *R_b.cub_* on the amount of MSP: 1—lower confidence level; 2—upper confidence level.

**Figure 6:**
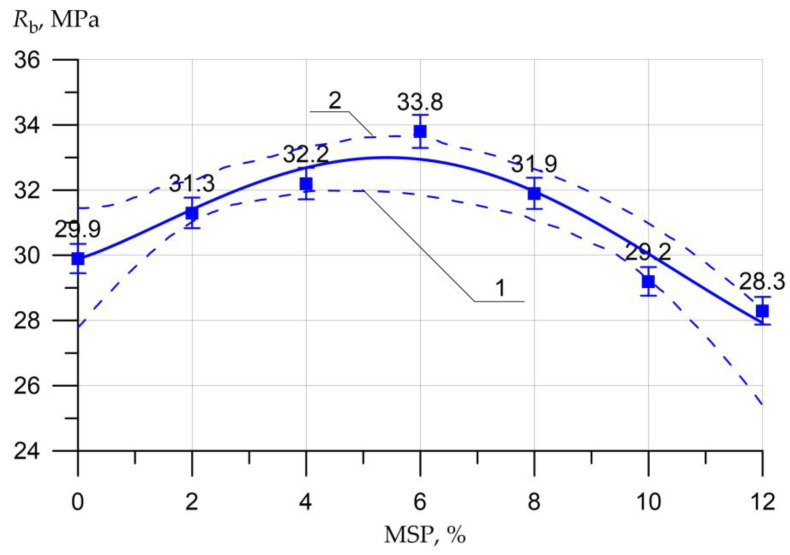
Dependence of the axial CS of concrete *R_b_* on the amount of MSP: 1—lower confidence level; 2—upper confidence level.

**Figure 7:**
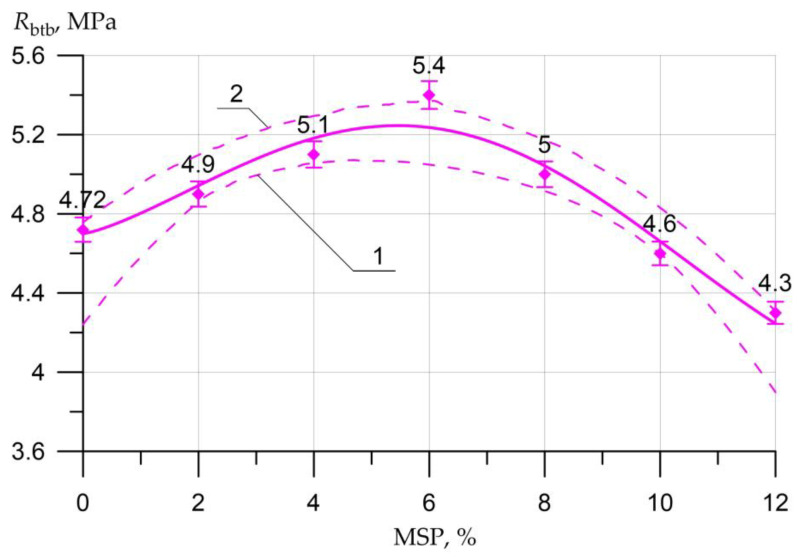
Flexural TS *R_btb_* of concrete as a function of the amount of MSP: 1—lower confidence level; 2—upper confidence level.

**Figure 8:**
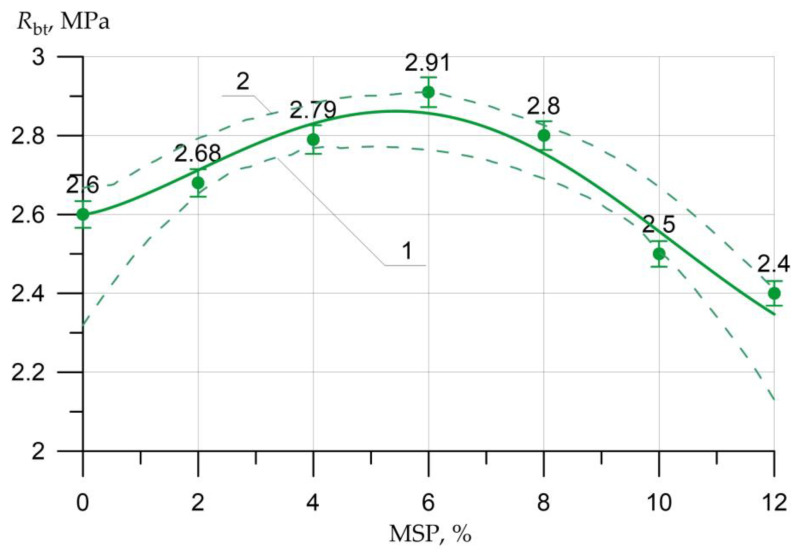
Dependence of the axial TS of concrete *R_bt_* on the amount of MSP: 1—lower confidence level; 2—upper confidence level.

**Figure 9:**
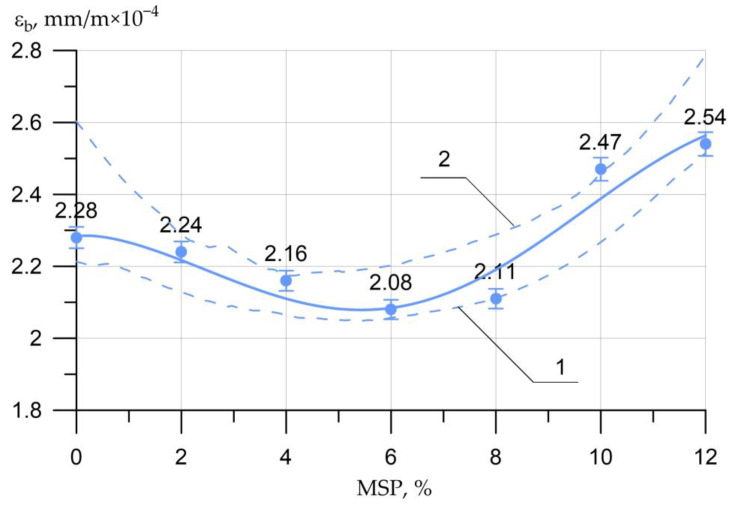
Effect of MSP dosage on ultimate strains in axial compression (USAC) *ε_b_*: 1—lower confidence level; 2—upper confidence level.

**Figure 10:**
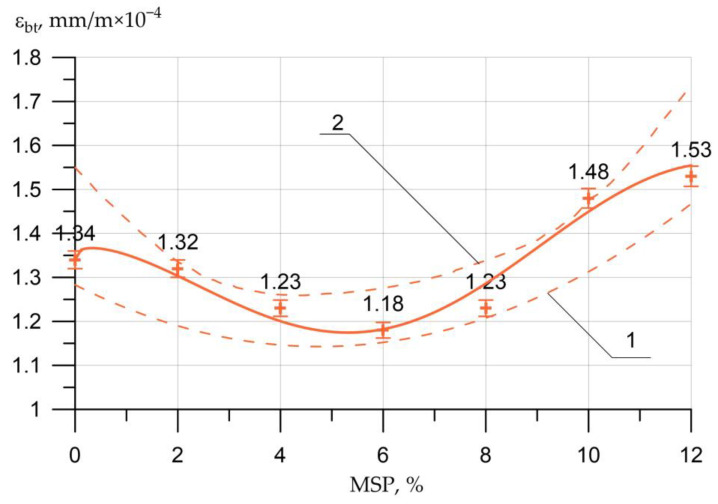
Effect of MSP dosage on ultimate strains in axial tension (USAT) *ε_bt_*: 1—lower confidence level; 2—upper confidence level.

**Figure 11:**
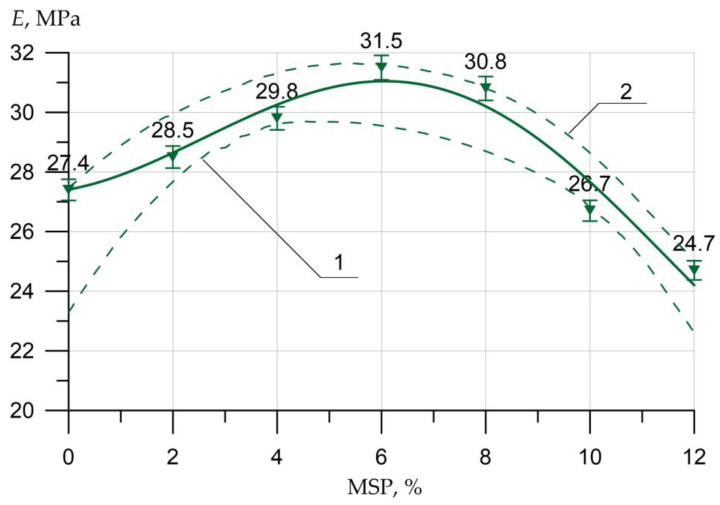
Dependence of the ME of concrete on the amount of MSP: 1—lower confidence level; 2—upper confidence level.

The results of the SSC of the studied concrete samples with a different proportion of MSP instead of a part of cement are presented in the form of graphs in Figure 5, Figure 6, Figure 7, Figure 8, Figure 9, Figure 10 and Figure 11.

Figure 5 shows the relationship between concrete strength (CS) *R_b.cub_* as determined by the cubed samples and MSP dosage.

According to the results of the study on concrete for compression, Figure 5 shows that the maximum strength value was demonstrated by samples with the replacement of cement with MSP in the amount of 6%, compared with the control sample, where the increase was 12%. When replacing cement with MSP in the amount of 2%, 4%, and 8%, the increments were smaller and amounted to 3%, 7%, and 8%, respectively. A drop in strength from 1% to 7% was observed when replacing the cement with MSP in an amount of 10–12%.

Figure 6 shows the relationship between axial CS, as determined by the prismed samples, and MSP dosage.

The dependence of the axial CS (Figure 6) on MSP looks similar to the CS. The axial CS maximum value when replacing part of the cement with MSP in the amount of 6% was 33.8 MPa, which is 13% higher than the strength of ordinary concrete. Additionally, the minimum value of the axial CS was recorded at a powder dosage of 12% and was equal to 28.3 MPa, which is 5% less compared to the strength of ordinary concrete.

Figure 7 shows the flexural strength versus MSP dosage.

Following the data presented in Figure 6, the highest flexural strength was observed in concrete with a 6% replacement of cement with MSP. The increase in TS in bending was 14%. An increase from 4% to 8% was also observed in samples with a dosage of MSP around the value of 2%, 4%, and 8%. The decrease in strength was observed in samples with a dosage of MSP at 10% and 12%-respectively, 3% and 9%.

Figure 8 shows the effect of the MSP dosage on axial TS (split tensile strength).

Figure 8 shows that the axial TS reached a maximum at a dosage of 6% MSP. The increase in strength at this dosage was 12%. An increase was also observed when replacing cement with MSP in the amount of 2%, 4%, and 8%, which is equal to 3%, 7%, and 8%, respectively. A similar trend in strength decline was observed when the dosage of MSP was more than 10%. The decline in strength was up to 8% at a dosage of MSP 12%.

The characteristics of the concrete strength *Y(x)* with the addition of MSP (*x* in all equations) were approximated by the “saturation” function [48] in the form:

(1) $$Y(x)=C_{0}+Ax^{b}\sin\left(\omega x+\varphi \right)$$

Here, *C*_0_ is characteristic of concrete without the addition of MSP; *A, b, ω, φ* are constants to be determined. 

Equations (2)–(5) show a high degree of closeness to the connection (determination coefficient $R^{2}$) of strength characteristics $R_{b.cub}$, $R_{b}$, $R_{btb}$, $R_{bt}$ with the content of MSP (in equations *x*)

(2) $$R_{b.cube}=\;42.3\;+\;1.536\;\times \;x^{0.607}\times \;\sin(0.296x\;+\;0.12),\;R^{2}=0.971$$

(3) $$R_{b}\;=\;\;29.9\;\;+\;2.28\times x^{0.185}\times \sin(0.31\;x+0.0),\;\;\;\;\;R^{2}=\;0.932$$

(4) $$R_{btb}=\;4.7\;+\;0.328\times x^{0.309}\times \sin(0.32x\;+\;0.0),\;\;\;\;\;\;R^{2}=0.952$$

(5) $$R_{bt}=\;\;2.6\;+\;0.143\times x^{0.370}\times \;\;\sin(0.327\;x\;+\;0.0),\;\;\;\;\;R^{2}=0.955$$

The dependences of the ultimate strains in compression (USAC) and in tension (USAT) are shown in Figure 9 and Figure 10, respectively.

The deformation characteristics were also approximated by the “saturation” function [48], which relates the ultimate deformations $\varepsilon_{b},\;\varepsilon_{bt}$ to the content of MSP *x* (6), (7)

(6) $$\varepsilon_{b}=\;2.28\;-\;0.0868\times x^{0.515}\times \sin(0.375\;x\;-\;0.218),\;\;\;\;\;R^{2}=0.923$$

(7) $$\varepsilon_{bt}=\;1.34\;-\;0.0974\times x^{0.323}\times \sin(0.425\;x\;-\;0.545),\;\;\;\;\;R^{2}=0.936$$

Changes in the values of USAC $\Delta \varepsilon_{b}$ and tension $\Delta \varepsilon_{bt}$ compared to the control composition are presented in Table 6.

Figure 9 and Figure 10 show that at the MSP dosage from 0% to 6%, compressive and tensile strains decreased and reached their lowest value at 6%, which is associated with an increase in strength characteristics. Further, at dosages of MSP from 8% to 12%, the deformations increased, which is associated with a drop in strength characteristics for a given range of the amount of the modifying additive.

The ME, depending on the content of the MSP, is well approximated by the saturation function (8):

(8) $$E=27.4\;+\;1.134\times x^{0.687}\times \sin(0.293\;x\;+\;0.163),\;\;\;\;\;\;\;\;R^{2}=0.951$$

Analyzing Figure 11, we can conclude that the maximum ME is observed when replacing the cement with MSP in an amount of 6%. The maximum value of the increase in ME was 15%. The increase in the elasticity modulus at powder dosages of 2%, 4%, and 8% was 4%, 9%, and 12%, respectively. Additionally, the drop in the elasticity modulus at a powder dosage of more than 10% ranged from 3% to 10%.

The graphs presented in Figure 5, Figure 6, Figure 7, Figure 8, Figure 9, Figure 10 and Figure 11 show the non-linear dependences of strength and deformability on the content of MSP. Concrete is a heterogeneous material, and the error in determining strength and deformability leads to significant dispersion when constructing confidence boundaries (shown by dotted lines at a confidence level of 0.95). A significant range of dispersion is due to the statistical dispersion of characteristics and non-linear dependence. For linear models, this dispersion is much smaller.

**Figure 12:**
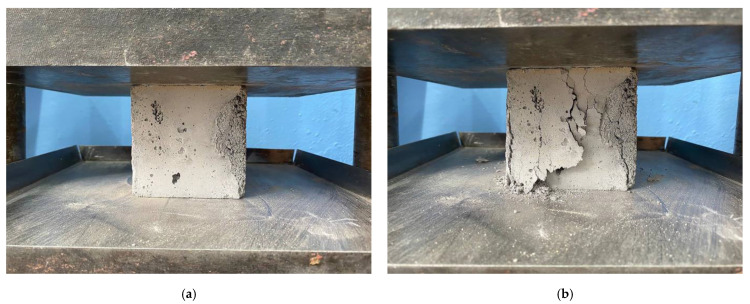
Photographs of the collapsed concrete specimen containing 6% MSP: (**a**) Initial phase; (**b**) Collapse phase.

Figure 12a,b shows the collapsed concrete specimens.

**Figure 13:**
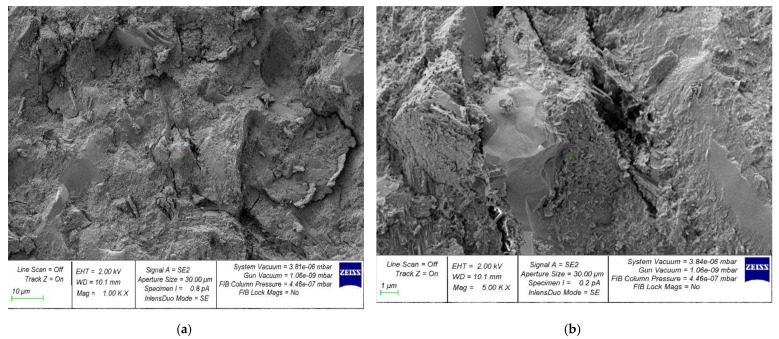
Samples containing 6% MSP: (**a**) 1000×; (**b**) 5000×.

**Figure 14:**
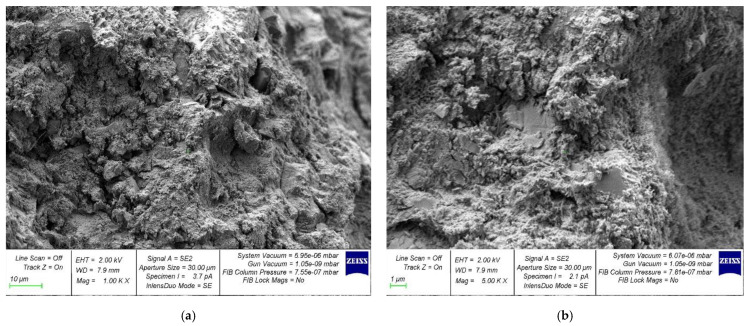
Samples of the control composition: (**a**) 1000×; (**b**) 5000×.

The microstructure photos of the hardened cement samples with 6% MSP and the control composition are presented in Figure 13 and Figure 14, respectively.

The SEM analysis data show that a concrete sample with MSP in an amount of 6% has the most complete microstructure, without voids and cracks (Figure 13) compared to a specimen of the control composition (Figure 14). The SEM microstructure of the samples is related to their mechanical properties, which is confirmed by the dependencies shown in Figure 5, Figure 6, Figure 7, Figure 8, Figure 9, Figure 10 and Figure 11. Additionally, the use of finely ground powder from mussel shells contributes to the additional formation of CSH gel zones, which in turn, ensures an increase in strength characteristics.

The mechanism of the formation of additional CSH-gel zones during the hydration of Portland cement can be explained as follows. The formation of semi-permeable shells on the surface of the initial cement particles is preceded by a long induction period, during which a solution supersaturated with Ca^++^ ions is created. After the formation of gel shells, the diffusion of calcium ions in the direction from the cement particle to the liquid phase continues at a relatively high rate, and after the saturation of the solution, the reverse diffusion process occurs, caused by a decrease in the concentration of Ca^++^ ions due to the formation of crystalline hydrates (in particular, hydrosilicate) in the thickness cement particles under the shell [49]. Due to the additional introduction of MSP, the amount of cement gel that is formed increases. As a result, a larger amount of gel-like substance is able to cover the remaining non-hydrated grains of cement clinker and increase the total volume of neoplasms in the hardened cement paste, which in turn, leads to an increase in the density and strength of the hardened cement paste, and also reduces porosity.

Thus, SEM analysis confirms the effectiveness of the adopted composition, technological solution, and the established rational dosage of MSP, which became possible due to a comparative analysis according to the criterion of structural integrity and the nature of microcracking, which cannot be established with the naked eye. The creation of a denser structure, the better packing of particles at the micro level due to modification, and the creation of additional crystallization centers make it possible to obtain a better, denser, and more durable structure of a new environmentally friendly concrete based on the finely ground powder of mussel shells.

**Figure 15:**
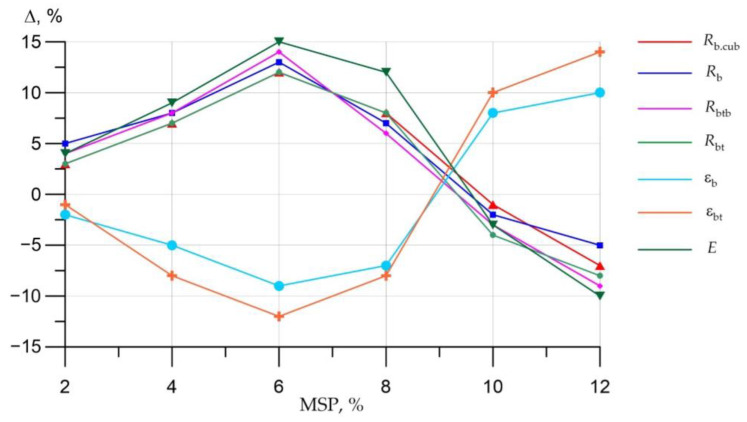
Curves of change in strength and deformation characteristics (SSC) in percentage terms compared to the control composition.

To achieve a more precise trend of the transformation of the characteristics of concrete related to the amount of MSP additive applied instead of cement, the nature of their change is presented in graphical form (Figure 15).

Figure 15 clearly demonstrates that the optimal values of SSC are fixed at a dosage of MSP in the amount of 6%. In this section of the horizontal axis, the maximum values of CS, axial CS, flexural TS, axial TS, and ME are observed, as well as the smallest values of USAC and USAT. Thus, the substitution of the cement part with 6% powder from mussel shells provides an increase in the above strengths and ME by 12%, 13%, 14%, 12%, and 15%, respectively, while the ultimate strain values decrease by 9% for USAC and by 12% for USAT. In general, the cement substitution with mussel powder by approximately 2–8% insured an increase in strength characteristics. This replacement range proved to be optimal. However, the replacement of cement with MSP over 10% provides a different effect, expressed in a decrease in strength characteristics and ME and in an increase in ultimate deformations. Thus, the maximum drop in strength characteristics was recorded when replacing cement with MSP in the amount of 12%. CS dropped by 7% compared to the control composition, axial CS by 5%, flexural TS by 9%, axial CS by 8%, and ME by 10%. As for USAC and USAT, they increased by 10% and 14%, respectively.

The improvement in the characteristics of concrete modified with MSP is explained by the fact that the good compatibility of the MSP shell powder instead of part of the cement in an amount of up to 10% contributes to the development and a more complete process of hydration reactions with the additional formation of CSH gel zones. This also agrees well with the chemistry of MSP. In addition, at the micro- and macro-levels, denser packing of particles is observed at an MSP dosage of 6%. It has been experimentally established and confirmed using SEM analysis that MSP in the proportion of 6% leads to the best organization of the concrete structure, which is confirmed by the best SSC of the studied concrete [48,50].

The results of the experimental studies are in good agreement with the results of similar experimental data by a number of other scientists [51,52,53,54]. So, for example, in [51], studies on the influence of oyster shells and on the durability characteristics of porous concrete were carried out, according to the results of which the authors found that replacing cement with oyster shell powder should not exceed a dosage of 20%. In [52], the effect of the partial substitution of cement with scallop shell powder on the mechanical and thermal characteristics of concrete was investigated. Similarly, as in the present study and in [52], the substitution of part of the cement with shell powder by up to 10% does not lead to a decrease in the values of the mechanical characteristics of concrete. Additionally, in the case of the present study, there is even an increase in performance by up to 15%. Additionally, in [53], the authors studied the application of powder from oyster shells in concrete technology as a replacement for a binder together with a number of other wastes, namely, the wastes of the cast and ground granulated blast-furnace slag. Thus, according to the research results, the use of the triple system “oyster shell powder-casting slag-ground blast-furnace granulated slag” as a replacement for cement by up to 20% improves strength characteristics and reduces porosity and permeability compared to the control samples. With regard to mussel shells, the use of mussel shell waste as a replacement for sand and cement in the concrete of offshore constructions was considered in [54]. According to the results of studies on the replacement of sand or cement with various proportions of mussel shells, no significant changes in the decrease in the resistance of concrete to erosional destruction, except for the case of replacing a large amount of sand (20%), were recorded. In all other cases (replacement of 5–10% sand and cement), the effect of mussel replacement on erosive degradation was insignificant.

The authors of [27,28,29,30] studied the effect of modifiers on other origins as a substitute for cement (microsilica, nanosilica, fly ash, granulated blast furnace slag, electric arc furnace dust, recycled concrete powder) and obtained similar results due to the fact that the modification mechanism allowed them to improve the process of structure formation, and, accordingly, the properties of the resulting concrete. Thus, taking this model of structure formation as a basis, we worked out our own hypothesis, which was confirmed in the course of experimental studies. Thus, the effectiveness of the modification of concrete with various types of finely dispersed powder additives has been confirmed.

In most works [12,14,36,38,54] similar to the present study, improvements in concrete characteristics were less (1–10%) or completely absent (comparable to the values of control samples). This was due to the fact that mussel shells were used either instead of part of a more durable aggregate or instead of part of a binder, but at the same time, the particle sizes, most likely, did not give the maximum result, which consisted of the densest packing of particles in the hardened cement paste. Therefore, the performance gains were smaller.

Discussing the nature of change in the deformation characteristics of the resulting concrete, it should be noted that the modification of concrete is a prescription technique that differs significantly in its result from other previously known methods for controlling the properties of concrete-dispersed fiber reinforcement [44]. This difference is explained by the fact that dispersed fibers give the concrete composite a more viscous character of destruction, influencing the increase in its deformability. By using modifiers, including aquaculture waste powders, we give the concrete a more high-strength character and increase the unity of the structure by creating additional crystallization centers. Thus, the deformability of such concrete is significantly reduced.

Thus, the operational efficiency of the proposed method for use in buildings and structures with increased requirements for the strength of concrete is confirmed.

The effectiveness of the use of powders from various types of aquaculture waste, including mussel shells, is explained by the higher content of calcium in it and the high dispersion of particles, which contribute to the hydration of cement due to its high activity and the dissolution of calcium, which is present in large quantities in the shells of seashells. 

## 4. Conclusions

In general, the obtained results of SSC for concrete allow us to draw the following main conclusions regarding this study:

(1) The modification of concrete with the finely ground powder of mussel shells makes it possible to enhance the structure formation and SSC, provided that the optimal dosage of such a modifier is observed.
(2) Replacing part of the cement in the concrete with mussel shell powder at an amount of 6% leads to better concrete performance values.
(3) Replacing cement with MSP by up to 10% does not adversely affect strength characteristics, while adding more than 10% MSP leads to a drop in strength characteristics.
(4) The modification of concrete with MSP at an amount of 6% provides an increase in CS by up to 12%, axial CS-up to 13%, TS in bending-up to 14%, axial TS-up to 12%, and ME-up to 15%. The addition of MSP in an amount from 0 to 6% gives a smooth increase in concrete strength and ME, while the addition of MSP from 6 to 12% leads to a sharp drop in concrete strength and ME.
(5) The modification of concrete with MSP at an amount of 6% provides a reduction in the ultimate deformations of compression and tension by up to 12%, and an increase in the dosage by 12% leads to an increase in deformations up to 14%.
(6) SEM analysis showed that concrete with 6% MSP has a more integral microstructure, without voids and cracks in comparison with the control sample, and also contributes to the additional formation of CSH gel zones, which ensures an increase in the strength characteristics of the composite.

The significance of the study is not only scientific but also applied. The environmental friendliness of the new concrete is to clean up the environment from polluting aquaculture waste and reduce the number of consumed resources and emissions by replacing some of the produced concrete components with a powder of natural origin. Economic efficiency is expressed in the reduction of the cost of new concrete compared to traditional ones by about 17% and the cost of building construction by up to 15% due to a decrease in the percentage of defects.

## Figures and Tables

**Table 1:** Review of the use of aquaculture modifiers in the manufacture of building composite materials.

Purpose of Waste	Reference	Effect of the Waste Type and Dosage on the Properties of the Developed Composite Materials
Modifier for asphalt	[16,17,18]	The use of seashell powder in [16] in asphalt pavement technology has a fairly good effect on the properties of this material. The resistance to high-temperature deformations is improved, the creep modulus increases, and the creep rate decreases. In [17], the authors studied the possibility of using fish scales as a modifying additive in asphalt in an amount from 4% to 16% by the weight of asphalt. It has been established that the use of this type of waste increases the “adhesion, viscoelastic properties, temperature susceptibility, and resistance to permanent deformation of asphalt” [17]. In [18], the use of fish scale powder in asphalt in similar dosages was also studied, and an improvement in the characteristics of the developed composite was also observed.
Additive in cement production	[19,20]	The “binary and ternary systems of cement with the addition of seashell powder” developed in the study [19] are more environmentally friendly, have the same compressive strength (CS), and can be successfully used as an analog of conventional Portland cement. A similar study was carried out in [20], according to the results of which it was found that the developed compositions of cement with shell powder are close to the same characteristics (“density, fineness, specific surface area, chemical composition, setting time, CS, and tensile strength (TS) in bending”) of conventional Portland cement without additives, which makes these types of cement competitive [20].
Replacement of gypsum in the production of refractory materials	[21]	It was found that replacing gypsum with scallop shell powder at 40% and 50% by weight increased the resistance of the developed refractory materials to the acid attack in the first three weeks. However, then it started to decline. As for the CS, it deteriorated significantly by up to 80% compared to gypsum.
Expansion admixture in cement mortar	[22]	In the process of cement hydration, the introduction of “calcined oyster shell powder increases the heat of hydration at the initial stage of the reaction due to the dissolution of CaO and the formation of Ca(OH)_2_” [22]. According to “the results of the study, it was found that the content of this additive in the amount of 3% is optimal” [22].
Binder replacement in heavy concrete technology	[23,24,25,26,27,28,29,30,31]	“Based on the results of experimental studies in [23], the authors found that the introduction of additional binders, such as oyster shell powder, foundry slag, and ground blast-furnace granulated slag”, makes it possible to obtain concretes with strength characteristics that are comparable to the values of control samples on ordinary Portland cement. In [24], the mechanical properties of concrete were studied when replacing cement with 5%, 10%, and 15% shell powder and replacing coarse aggregate with 10%, 20%, and 30% crushed seashells. In general, following the results of this investigation, the optimal limits for replacing shell waste in concrete were established: for cement–5% and for coarse aggregate–10%. In [25], the mechanical behavior of high-strength concrete with the addition of shell powder at elevated temperatures was studied. So, according to the results of the study, the samples modified with shells “showed a higher CS and modulus of elasticity than the control sample. According to the results of the study, it was found that the use of shell ash powder in an amount of 5% makes it possible to obtain concretes with improved durability and strength” [26]. Replacing part of the cement with industrial waste (microsilica/nanosilica [27], fly ash [28], blast furnace granulated slag and electric arc furnace dust [29], and recycled concrete powder [30]) with certain formulation methods, leads to an improvement in mechanical, thermal, and wear-resistant properties and makes it possible to obtain high-strength environmentally efficient concrete [31]
Binder component in geopolymer solution	[32,33]	The shell waste powder was used in [32] as a precursor for the preparation of an alkali-activated mortar, making it possible to obtain a mortar “with a CS of 22 MPa and a porosity of 16.5%” [32]. In [33], the authors investigated the effect of replacing a part of metakaolin with powder from scallop shells. “Replacing part of the metakaolin with shell powder” [33] makes it possible to obtain geopolymer mortars with higher mechanical strength, lower porosity, and shrinkage than similar metacolin-based mortars.
Aggregate	[15,34]	In [34], to create permeable concrete, natural aggregate was replaced with crushed seashells in an amount of 60% of the weight of the natural aggregate. Following the results of these experiments, the developed permeable concrete had lower frost resistance and “strength compared to the control composition” [34]. “The use of seashells as a fine aggregate” was studied in [15]. The authors found the use of sand from crushed seashells in self-compacting concrete by up to 100% to be acceptable and without violating the basic properties. Small drops in the values of CS and elasticity modulus were recorded only at a replacement of 100%.

**Table 2:** Raw material characteristics for the CMs manufacture.

Portland Cement (PC) without Additives CEM I 52.5 N (Novoroscement, Novorossiysk, Russia)	Granite Crushed Stone (CrS) (South-Nerud, Pavlovsk, Russia)	Quartz Sand (QS) (“Arkhipovsky quarry”, Village Arkhipovskoe, Russia)
The normal density of cement paste is 25%. Passage through a sieve with a mesh size of 0.08 mm−96%. TS in bending-7.7 MPa, CS-56.2 MPa	Particle size 5–10 mm. Bulk density (BD)-1486 kg/m^3^. True density (TD)-2630 kg/m^3^. Crushability-12.1 wt.%. The content of needle-shaped grains is 6.2 wt.%	Fineness modulus-1.86. BD-1438 kg/m^3^. TD-2667 kg/m^3^. The content of dust and clay particles is 0.9%. Clay content in lumps-0.2%

**Table 3:** CC of mussel shells.

Element	O	C	Ca	Na	S	Mg	Si	Al
wt.%	35.1	11.7	51.8	0.3	0.2	0.2	0.4	0.3

**Table 4:** Composition of the CM.

Initial Materials	Actual Value
W/B	0.5
PC, kg/m^3^	380
W, L/m^3^	192
CrS, kg/m^3^	1261
QS, kg/m^3^	714

Note: W–water.

**Table 5:** Results of determining the actual density of concretes of all experimental compositions.

Concrete Characteristic	MSP, %						
	0	2	4	6	8	10	12
Average density (kg/m^3^)	2370	2373	2378	2381	2372	2365	2355

**Table 6:** Changes in USAC and USAT.

MSP, %	0	2	4	6	8	10	12
∆ε_b_, %	0	–2	–5	–9	–7	8	11
∆ε_bt_, %	0	–1	–8	–12	–8	10	14

## Data Availability

The study did not report any data.

## Abbreviations

BD	Bulk Density
CC	Chemical Composition
CM	Concrete Mixture
CS	Compressive Strength
CrS	Crushed Stone
ME	Modulus of Elasticity
MSP	Mussel Shell Powder
PC	Portland Cement
PSD	Particle Size Distribution
QS	Quartz Sand
SSC	Strength and Deformation Characteristics
TD	True Density
TS	Tensile Strength
USAC	Ultimate Strains in Axial Compression
USAT	Ultimate Strains in Axial Tension
